# Oroxylin A From *Scutellaria baicalensis* Improved Pulmonary Fibrosis of Radiation Pneumonia Through Ferroptosis by Nrf2

**DOI:** 10.1155/carj/4397833

**Published:** 2026-01-06

**Authors:** Wei Zhang, Wen Hao, Xiaoxia Gao, Kuo Li

**Affiliations:** ^1^ Department of Oncology and Hematology, The Affiliated Traditional Chinese Medicine Hospital, Southwest Medical University, Luzhou, 646000, China, swmu.edu.cn; ^2^ Department of Oncology, The Affiliated Traditional Chinese Medicine Hospital, Southwest Medical University, Luzhou, 646000, China, swmu.edu.cn

**Keywords:** ferroptosis, Oroxylin A, pulmonary fibrosis, radiation pneumonia

## Abstract

Radiation pneumonitis can result in respiratory dysfunction and progress to radiation fibrosis, and in severe cases, respiratory failure may occur. Hence, we investigated the effects of Oroxylin A from *Scutellaria baicalensis* in radiation pneumonia (RP) and the underlying mechanism. Oroxylin A improved pulmonary fibrosis, reduced oxidative stress, and promoted cell growth in model of RP. Oroxylin A suppressed ferroptosis and induced Nrf2 protein expressions in liver cells of model of RP. Moreover, Nrf2 inhibitor reduced the effects of Oroxylin A on pulmonary fibrosis in model of RP by oxidative stress. Collectively, these data suggest that Oroxylin A from *Scutellaria baicalensis* improved pulmonary fibrosis of RP through ferroptosis by Nrf2 axle.

## 1. Introduction

Radiation pneumonia (RP) is an important treatment modality for lung cancer [1]. During RP, normal lung tissue can be damaged due to radiation exposure, leading to the development of radiation pneumonitis with an incidence rate ranging from 14% to 49% [2]. Radiation pneumonitis can result in respiratory dysfunction and progress to radiation fibrosis, and in severe cases, respiratory failure may occur [1, 3]. This can lead to treatment interruption and, in extreme cases, indirect or direct patient mortality. However, limiting the radiation dose has adverse effects on disease control and prognosis [4].

Current research suggests that RP‐induced lung injury is a result of multiple cellular and molecular interactions, involving excessive accumulation, proliferation, and differentiation of fibroblasts, leading to excessive extracellular matrix (ECM) deposition and ultimately resulting in lung fibrosis [5]. With advancements in radiation therapy techniques and equipment, the incidence of adverse reactions caused by RP has significantly reduced [6, 7]. The specific mechanisms underlying radiation pneumonitis are not yet fully understood, and the traditional approach of evaluating RP plans based on mean lung dose remains a major criterion [8].

As a routine treatment for malignant tumors, radiotherapy has been widely used in the treatment of thoracic solid tumors [9]. However, while radiation kills tumor cells, it also causes damage or even death to normal cells present in the same part of the human body due to its lack of discernment function, including radiation‐induced skin damage and radiation‐induced lung injury [10]. If the degree of radiation damage is severe, it not only directly affects the effectiveness of radiotherapy but also interrupts the radiotherapy plan, affects the effectiveness of tumor treatment, and in severe cases, can induce pulmonary ventilation dysfunction, severe pulmonary fibrosis, respiratory failure, and even death in patients [11]. Epithelial–mesenchymal transition (**EMT**) is one of the important pathways through which mesenchymal cells produce and modulate **ECM** and contribute to its excessive secretion [12].

Ferroptosis is an important factor in pulmonary fibrosis caused by RP [13]. Iron death has been proved to be related to cancer‐related cell death, ischemia‐reperfusion injury, neurological diseases, and acute kidney injury [14–16]. This change in mitochondrial structure is a morphological marker of iron death and helps to distinguish it from apoptosis (chromatin condensation, cytoskeleton rupture, and apoptotic body formation), and necrotic apoptosis [17–22]. In genetics, iron death is associated with multiple iron metabolism‐related genes, lipid synthesis, and oxidative stress pathways [23]. Furthermore, studies have found a connection between ferroptosis and pulmonary fibrosis caused by RP through the confirmation of certain compounds and traditional Chinese medicines [24].

Nuclear factor‐erythroid 2‐related factor‐2 (Nrf2) signaling pathway is highly correlated with the mechanism of ferroptosis regulation [25]. Upon nuclear translocation, Nrf2 promotes the transcription of multiple genes in preventing ferroptosis, including cystine/glutamate transporter, ferritin light and heavy chains, and even glutathione peroxidase 4 (GPX4) itself, all of which are target genes of Nrf2 [26]. This indicates that regulation of Nrf2 is an important pathway for preventing ferroptosis in pulmonary fibrosis caused by RP [27, 28].

*Scutellaria baicalensis*, also known as Huangqin, contains various chemical components, including flavones, flavonoid glycosides, polysaccharides, volatile oils, and trace elements [29]. The most important constituents are flavones and flavonoid glycosides [30]. Huangqin has a bitter taste and a cold property, and it exhibits pharmacological effects such as heat‐clearing, dampness‐drying, fire‐purging, detoxification, hemostasis, and fetus‐stabilizing. Huangqin possesses antimicrobial, antitumor, antiviral, hypoglycemic, hepatoprotective, neuroprotective, cardioprotective, and immunomodulatory effects [31–33]. Oroxylin A is the main active compound in Huangqin and exhibits various pharmacological effects [34–36]. Hence, we investigated the effects and underlying mechanism of Oroxylin A from *Scutellaria baicalensis* in pulmonary fibrosis caused by RP.

## 2. Materials and Methods

### 2.1. Patients With RP

RP was diagnosed and collected from our hospital. This study was approved by the Ethics Committee of our hospital. Informed consent was obtained from all participants (2023‐131).

### 2.2. In Vivo

C57BL/6J mice were intraperitoneally injected with pentobarbital sodium (40 mg/kg). Under anesthesia, mice were performed using the computed tomography guided SmART irradiator (Precision X‐ray, Madison, CT). A circular 15‐mm treatment field was centered on the right lung. Five fractions of 9 Gy were given once daily, with 2 equally weighted opposed parallel beams (225 kVp, 20 mA, 0.32 mm Cu filter [0.89 HVL], 4.26 Gy/min, beam angles 30° and 210°) [37]. This study was approved by the Ethics Committee of our hospital (20,221,217,146). The low group of mice was given 10 mg/kg once a day of Oroxylin A (i.p.), the Med group of mice was given 20 mg/kg once a day of Oroxylin A (i.p.), and the high group was given 40 mg/kg once a day of Oroxylin A (i.p.) [38].

### 2.3. Cell Culture and Proliferation Assay

A549 cells were also cultured under linac with an 8‐Gy dosage; the medium was replaced with serum‐free medium 2 h before irradiation [37]. After culturing at indicated time, the cellular proliferation was detected using 3‐(4,5‐dimethylthiazol‐2‐yl)‐2,5‐diphenyltetrazolium bromide (MTT) assay according to manufacturer’s instructions.

## 3. Quantitative Polymerase Chain Reaction (qPCR) and Enzyme‐Linked Immunosorbent Assay

qPCR was performed with the ABI Prism 7500 sequence detection system according to the Prime‐ScriptTM RT detection kit. Relative levels of the sample mRNA expression were calculated and expressed as 2^−ΔΔCt^. Inflammatory factors and oxidative stress were detected by using the IL‐1β, IL‐6, TNF‐α, reactive oxygen species (ROS), MDA, SOD, GSH, and GSH‐px Quantikine ELISA kit (PI301, PI326, PT512, S0033S, S0131S, S0101S, S0057S, S0056, Beyotime).

### 3.1. Western Blot

The membranes were incubated with primary antibodies: Nrf2 (1:1000, Abcam), GPX4 (1:1000, Abcam), and *β*‐actin (1:5000, Santa Cruz Biotechnology) after blocking with 5% BSA in TBS, followed by incubation with peroxidase‐conjugated secondary antibodies (Santa Cruz Biotechnology).

### 3.2. Electron Microscopy

Electron microscopy was executed according to the previously described method [39] using a Hitachi H7650 transmission electron microscope (Tokyo, Japan).

### 3.3. Statistical Analyses

*p* < 0.05 was considered statistically significant using GraphPad Prism 6. Comparisons of data between groups were followed using Student’s *t*‐test or one‐way analysis of variance (ANOVA), followed by Tukey’s post hoc test.

## 4. Results

### 4.1. Screening the for an Oroxylin A From *Scutellaria baicalensis* for New Nrf2 Agonists

To seek out new Nrf2 agonists, luciferase reporter assay was employed to screen small‐molecule compounds from an *Scutellaria baicalensis* drug (Figure 1(a)). A549 cells were transfected with Nrf2‐responsive luciferase plasmid, the promoter of which contains eight repeated Nrf2‐DNA binding motifs (8× GTIIC) (Figure 1(a)). Oroxylin A was identified to increase Nrf2 luciferase activity in A549 cells (Figure 1(b)). Oroxylin A (25–500 μM) reduced cell viability; Oroxylin A (0–125 μM) did not affect cell viability in A549 cells (Figure 1(c)). Meanwhile, Oroxylin A induced Nrf2 protein expression in A549 cells (Figure 1(d)).

Figure 1Screening for an Oroxylin A from *Scutellaria baicalensis* for new Nrf2 agonists (a), luciferase activity level (b), cell viability (c), and Nrf2 protein expression (d). ^∗∗^ *p* < 0.01 compared with the DMSO group.

**Figure:**
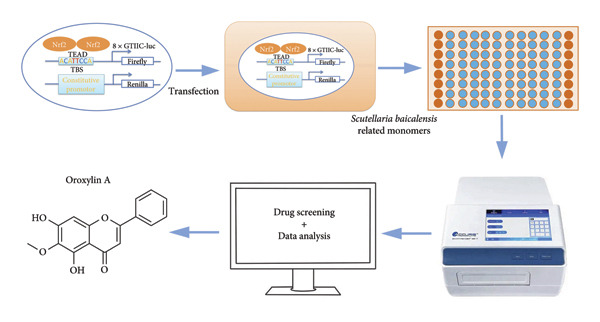
(a)

**Figure:**
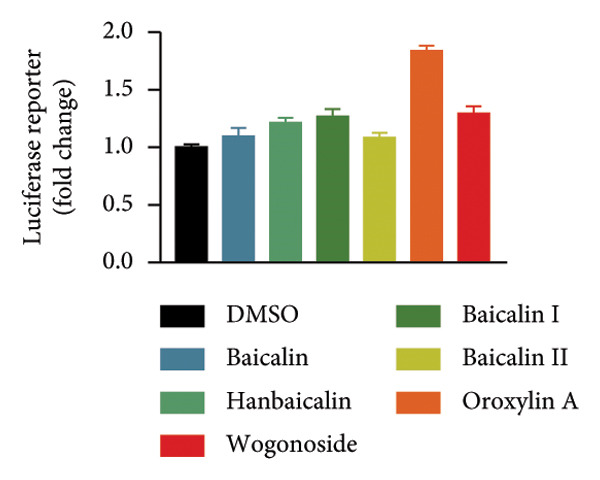
(b)

**Figure:**
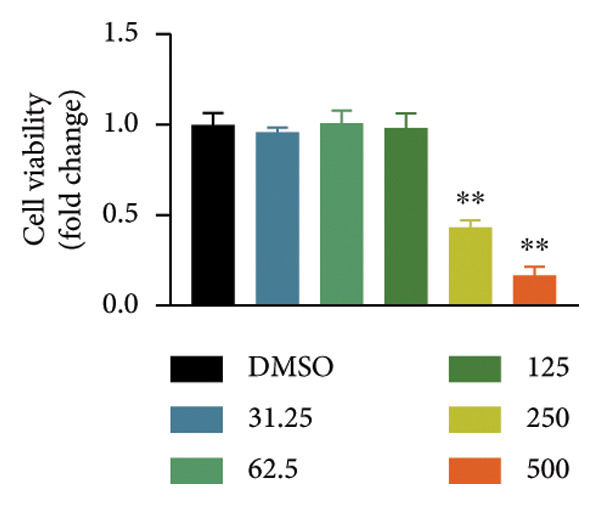
(c)

**Figure:**
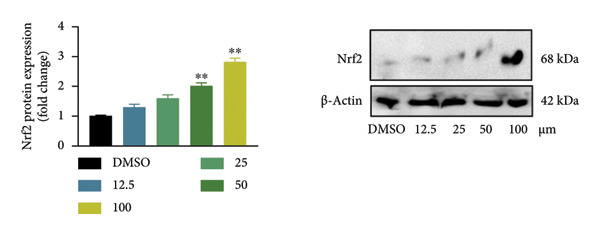
(d)

### 4.2. Oroxylin A Induced Nrf2 Protein Expression

In addition, we seek out the function of Oroxylin A on Nrf2 protein expression in model of pulmonary fibrosis of RP. Oroxylin A induced Nrf2 protein expression in the mice model or in the in vitro model of RP (Figures 2(a), 2(b)). Drug and protein linkage analysis showed that Oroxylin A is linked to the Nrf2 protein (Figure 2(c)). Nrf2 protein expression at 16‐ARG and 74‐ASN changed at 16‐ALA and 74‐ALA (Figure 2(d)). CETSA with HEK293T cells demonstrated that Oroxylin A largely improved the thermal stability of exogenous WT‐Nrf2, while CA did not change the thermal stability of Mut‐Nrf2, indicating 16‐ALA and 74‐ALA might be responsible for the interaction between Nrf2 and Oroxylin A (Figures 2(e), 2(f)).

Figure 2Oroxylin A induced Nrf2 protein expression. Nrf2 protein expression in the mice model (a) and in the in vitro model (b), drug and protein linkage analysis (c), 16‐ALA and 74‐ALA of Nrf2 protein expression (d), CETSA for the thermal stability (e, f). ^##^ *p* < 0.01 compared with the sham or control group.

**Figure:**
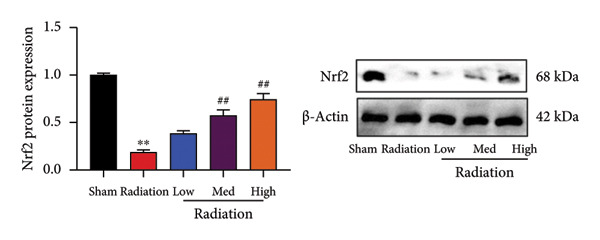
(a)

**Figure:**
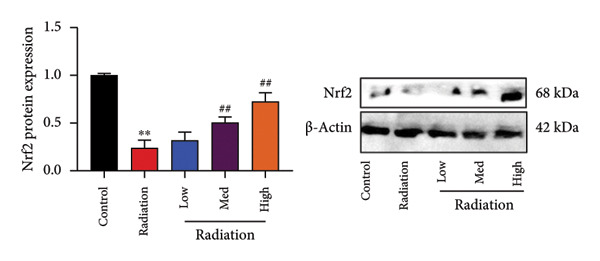
(b)

**Figure:**
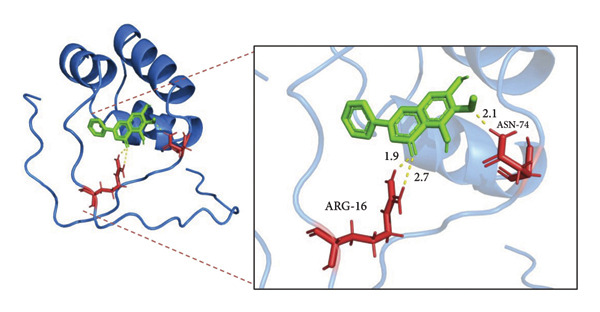
(c)

**Figure:**
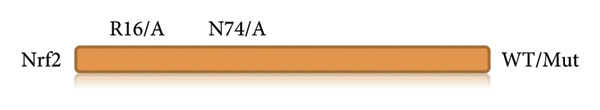
(d)

**Figure:**
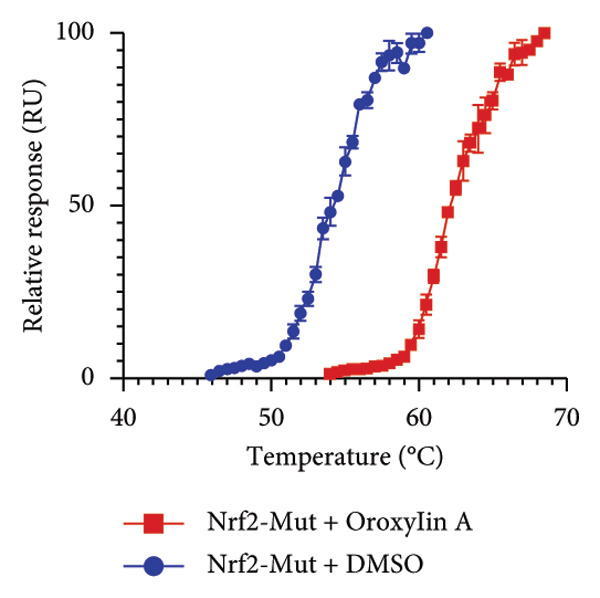
(e)

**Figure:**
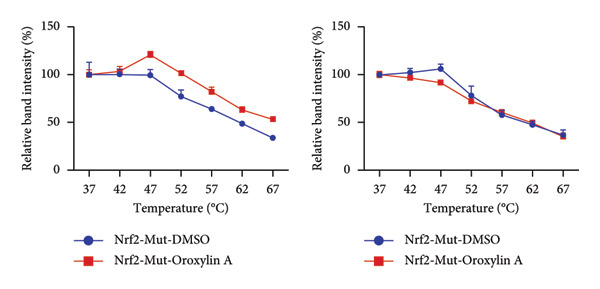
(f)

### 4.3. Oroxylin A Improved Pulmonary Fibrosis of RP in Mice Model

We further explored whether Oroxylin A affected on pulmonary fibrosis of RP. Oroxylin A reduced inflammatory score and bronchiolar epithelium thickness, inhibited airway construction and central airway resistance, improved pulmonary fibrosis, increased inspiratory capacity and quasi‐static compliance, and suppressed IL‐1β and IL‐6 activity levels in mice model of RP (Figure 3).

Figure 3Oroxylin A improved pulmonary fibrosis of radiation pneumonia in the mice model. Inflammatory score (a), bronchiolar epithelium thickness (b), pulmonary fibrosis (c), airway construction (d), central airway resistance (e), inspiratory capacity (f), quasi‐static compliance (g), and IL‐1β and IL‐6 activity levels of lung tissue in the mice model (h, i). ^∗∗^ *p* < 0.01 compared with the sham group, ^##^ *p* < 0.01 compared with the radiation group.

**Figure:**
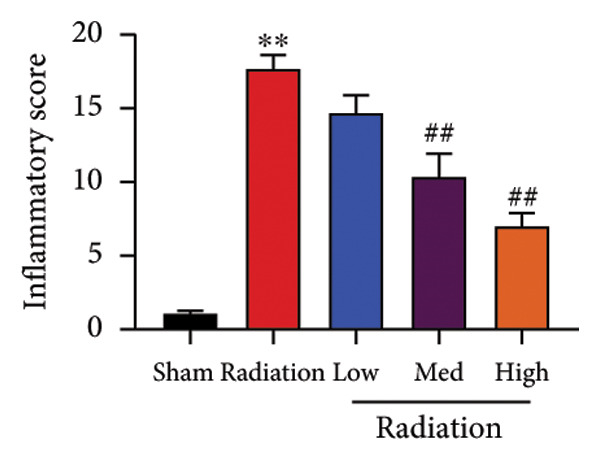
(a)

**Figure:**
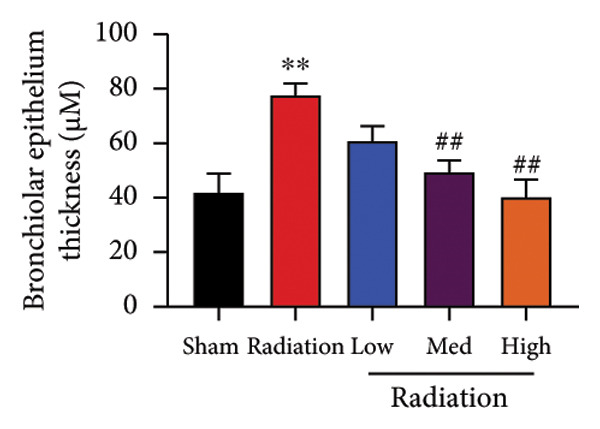
(b)

**Figure:**
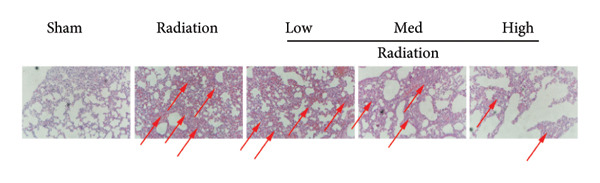
(c)

**Figure:**
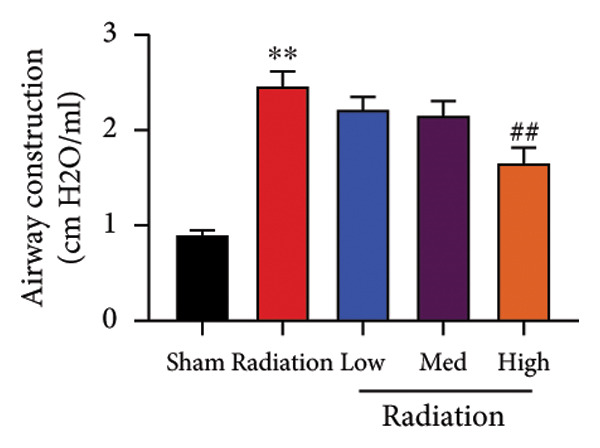
(d)

**Figure:**
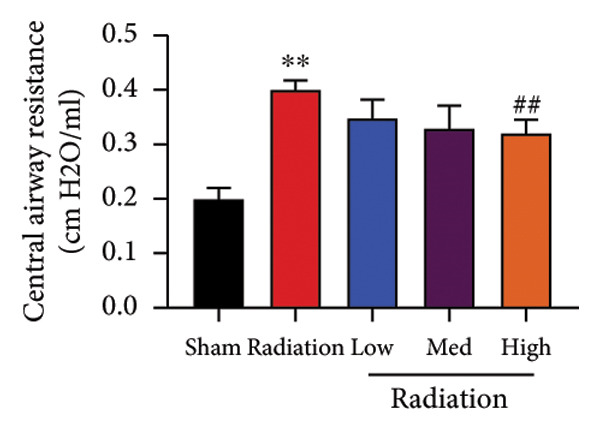
(e)

**Figure:**
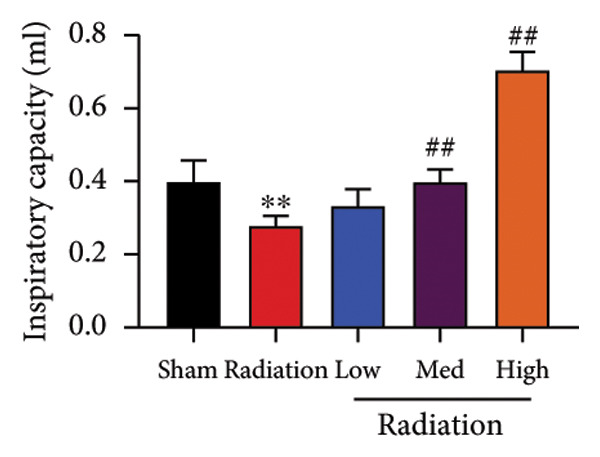
(f)

**Figure:**
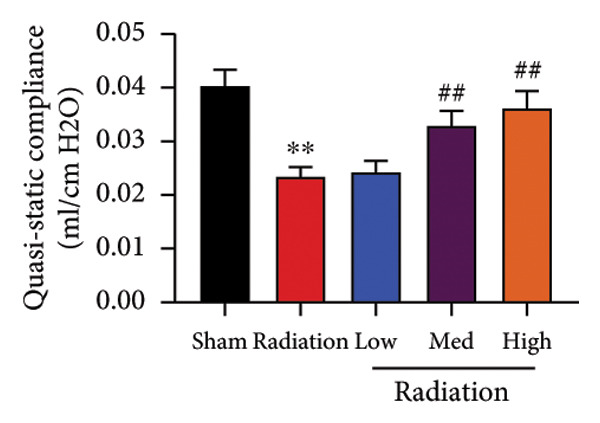
(g)

**Figure:**
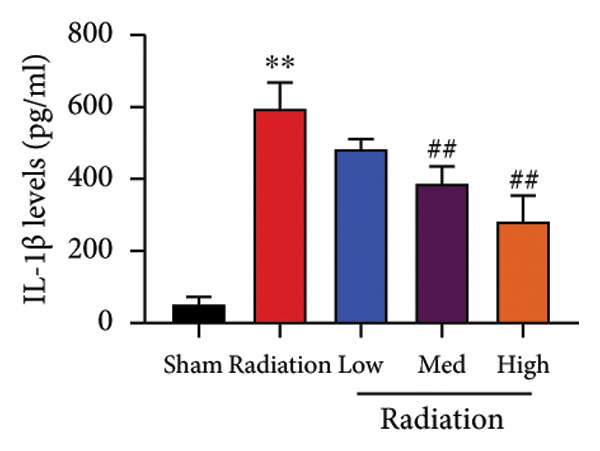
(h)

**Figure:**
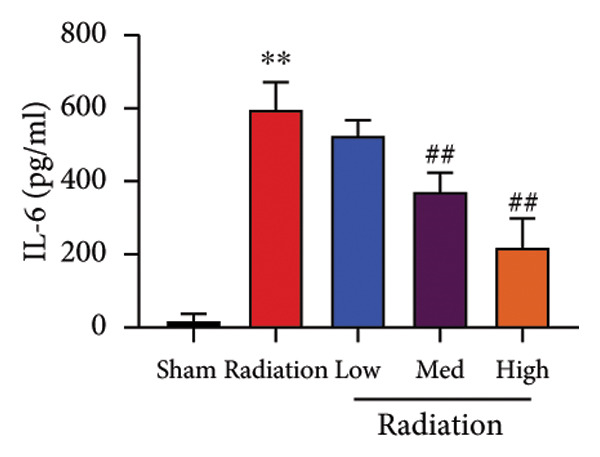
(i)

### 4.4. Oroxylin A Reduced Inflammation and Oxidative Stress in the Mice Model or in the In Vitro Model

The study examined the effects of Oroxylin A on inflammation and oxidative stress in the mice model or in the in vitro model. Oroxylin A reduced TGF‐β and MDA levels, increased SOD and GSH activity levels, and suppressed col3a1, FN, SMA, and twist mRNA expressions in lung tissue of mice model of RP (Figure 4). Oroxylin A suppressed IL‐1β, IL‐6, and TNF‐α levels, reduced ROS and MDA levels, increased SOD and GSH‐PX levels, and suppressed col3a1, FN, SMA, and twist mRNA expressions in the in vitro model of RP (Figure 5).

Figure 4Oroxylin A reduced inflammation and oxidative stress in the mice model. TGF‐β and MDA levels (a, b), SOD and GSH activity levels (c, d), and col3a1/FN/SMA/twist mRNA expressions of lung tissue in the mice model (e, f, g, h) ^∗∗^ *p* < 0.01 compared with the sham group, ^##^ *p* < 0.01 compared with the radiation group.

**Figure:**
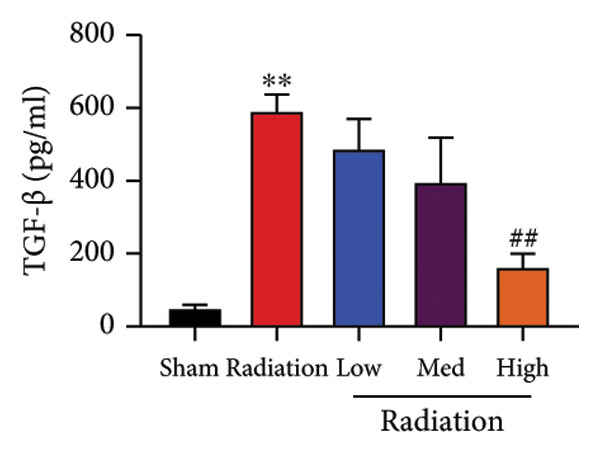
(a)

**Figure:**
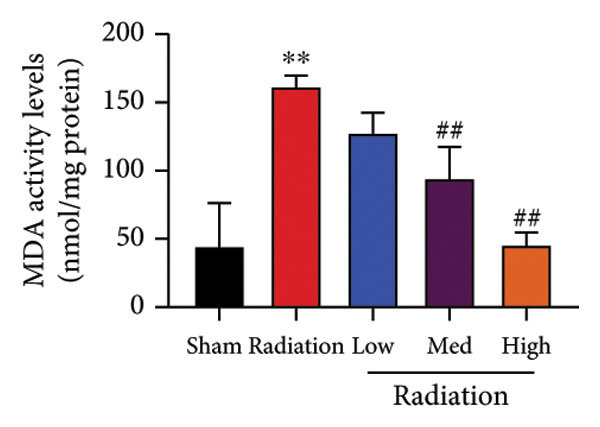
(b)

**Figure:**
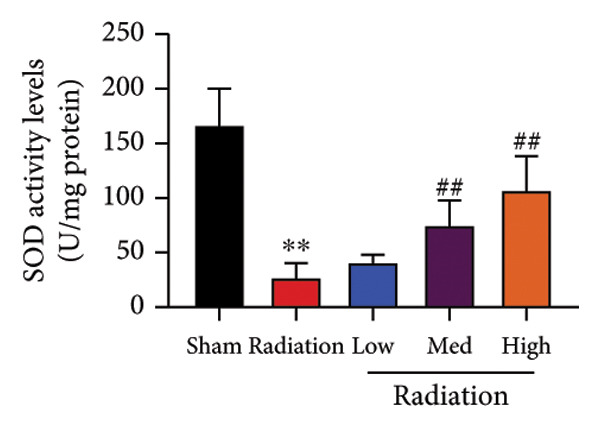
(c)

**Figure:**
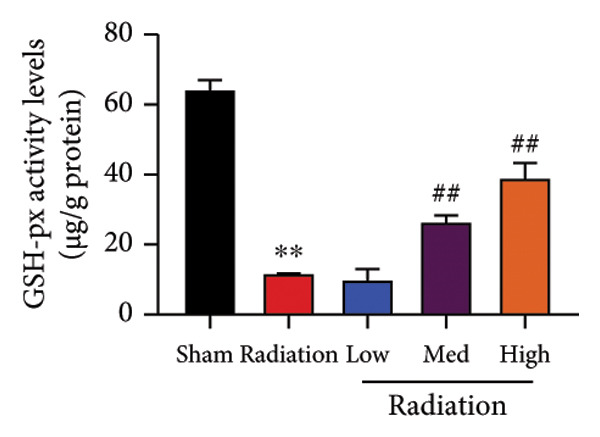
(d)

**Figure:**
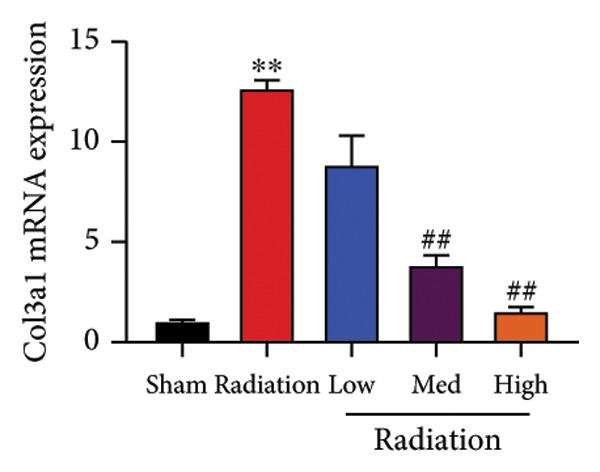
(e)

**Figure:**
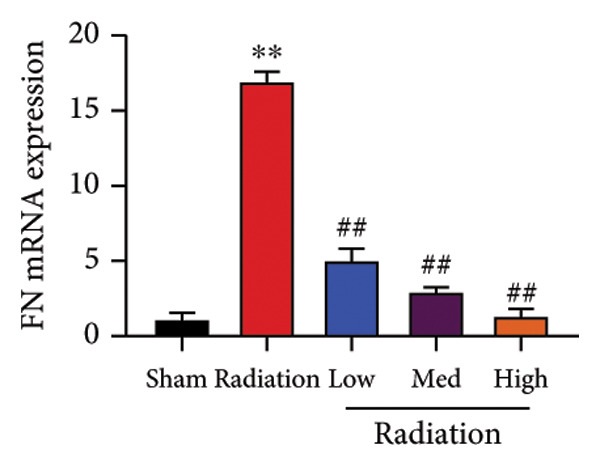
(f)

**Figure:**
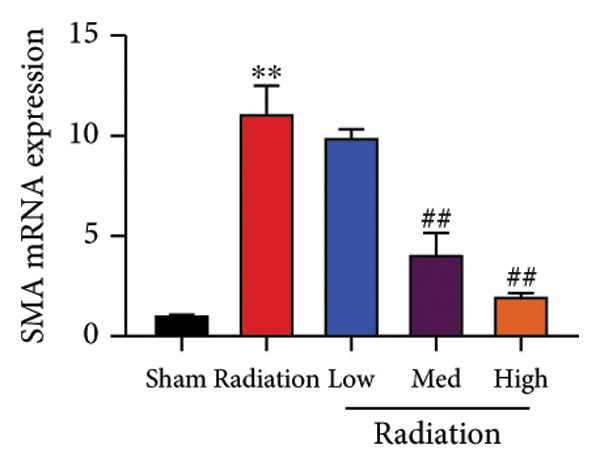
(g)

**Figure:**
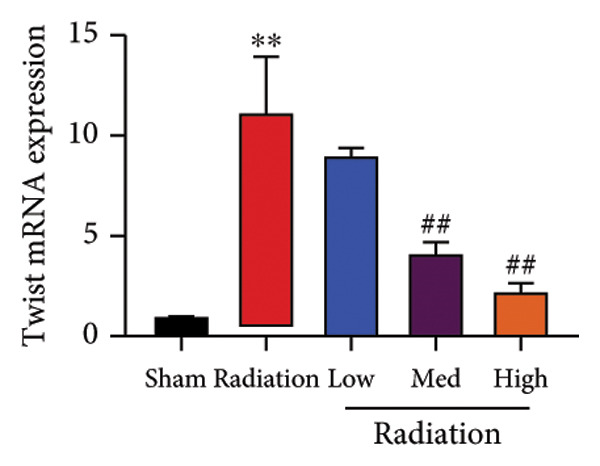
(h)

Figure 5Oroxylin A reduced inflammation and oxidative stress in the in vitro model. IL‐1β/IL‐6/TNF‐α (a), MDA/SOD/GSH‐PX/ROS (b), and col3a1/FN/SMA/twist mRNA expressions in the in vitro model (c, d, e, f). ^∗∗^ *p* < 0.01 compared with the control group, ^##^ *p* < 0.01 compared with the radiation group.

**Figure:**
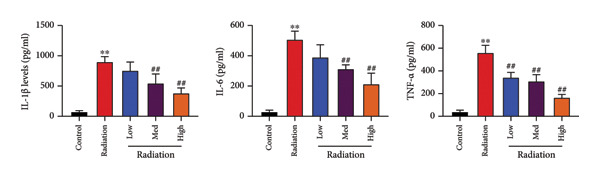
(a)

**Figure:**
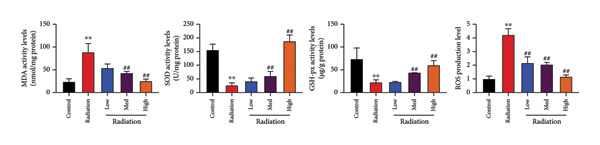
(b)

**Figure:**
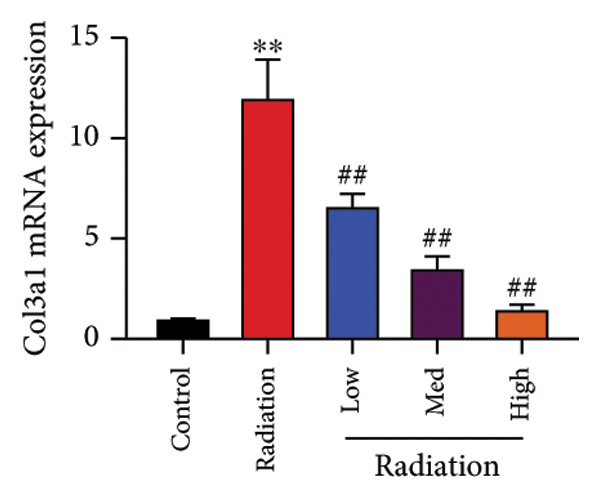
(c)

**Figure:**
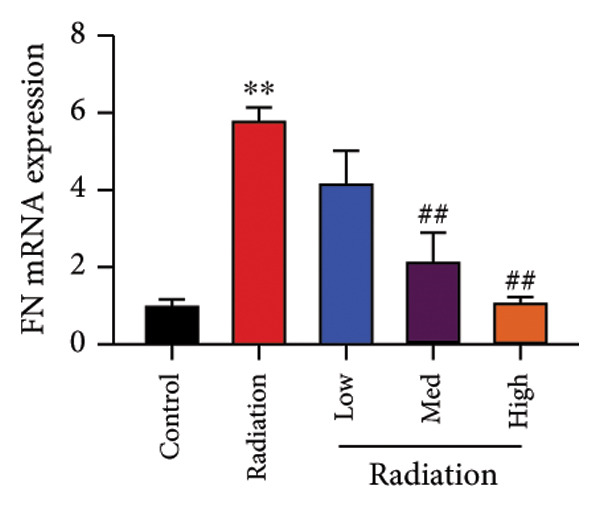
(d)

**Figure:**
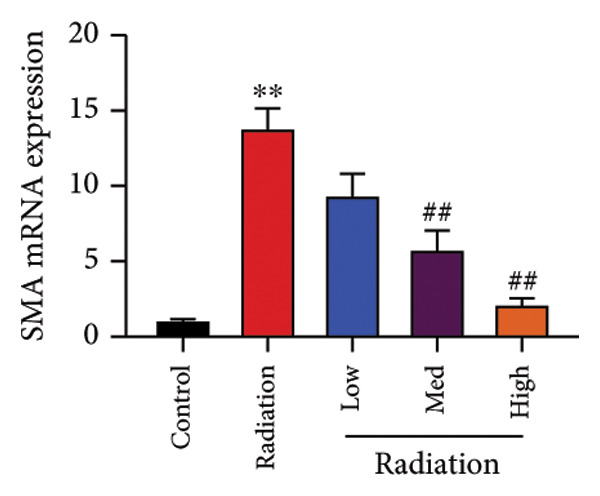
(e)

**Figure:**
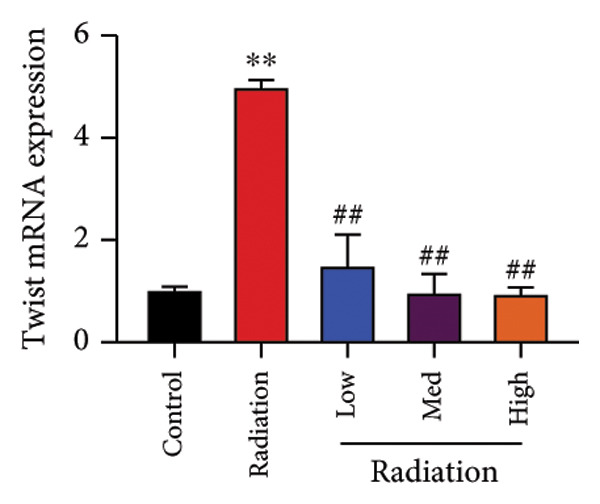
(f)

### 4.5. Oroxylin A Reduced Ferroptosis in the Mice Model or in the In Vitro Model

To determine the effects of Oroxylin A on pulmonary fibrosis of RP, we analyzed the effects of Oroxylin A on ferroptosis in model of RP. Oroxylin A increased cell viability and reduced LDH activity, PI rate levels, and iron contents in the in vitro model of RP (Figures 6(a), 6(b), 6(c), 6(d)). Meanwhile, Oroxylin A reduced GSH and GPX4 protein expressions levels in the mice model or in the in vitro model of RP (Figures 6(e), 6(f), 6(g), 6(h)). Then, Oroxylin A increased JC‐1 and AM/Cocl2, and reduced mitochondrial damage in the in vitro model of RP (Figures 6(i), 6(j), 6(k)).

Figure 6Oroxylin A reduced ferroptosis in the mice model or in the vitro model. Cell viability (a), LDH activity (b), PI rate levels (c), iron contents (d), GSH activity (e), GPX4 protein expressions (f) in the in vitro model; GSH activity (g) and GPX4 protein expressions (h) of lung tissue in the mice model; JC‐1 and AM/Cocl2 (i, j) and mitochondrial damage (k) in the in vitro model. ^∗∗^ *p* < 0.01 compared with the sham or control group, ^##^ *p* < 0.01 compared with the radiation group.

**Figure:**
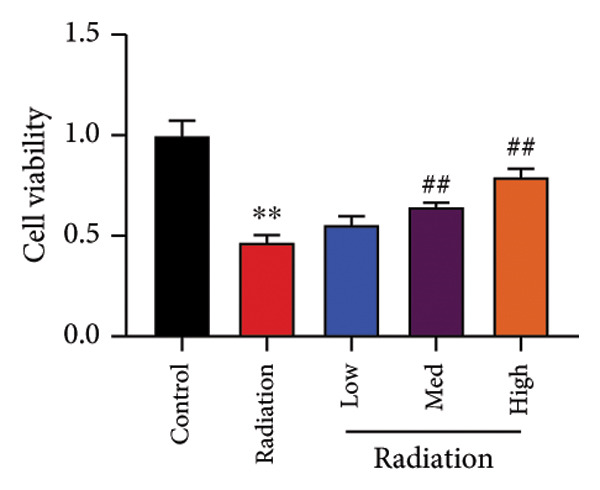
(a)

**Figure:**
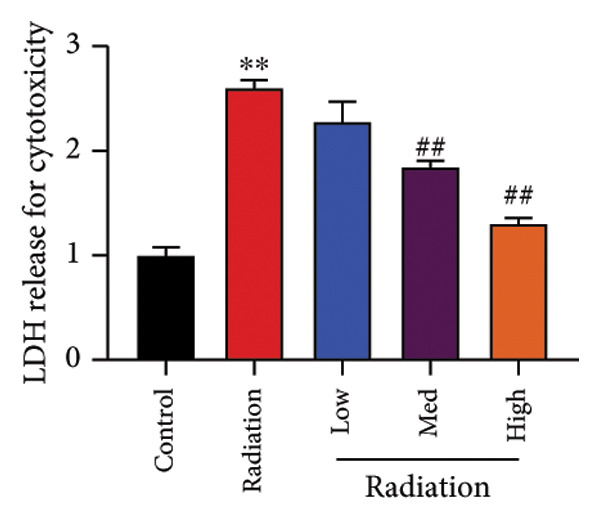
(b)

**Figure:**
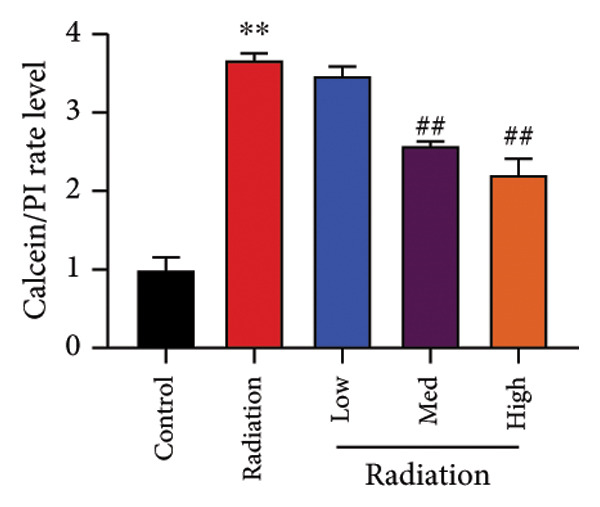
(c)

**Figure:**
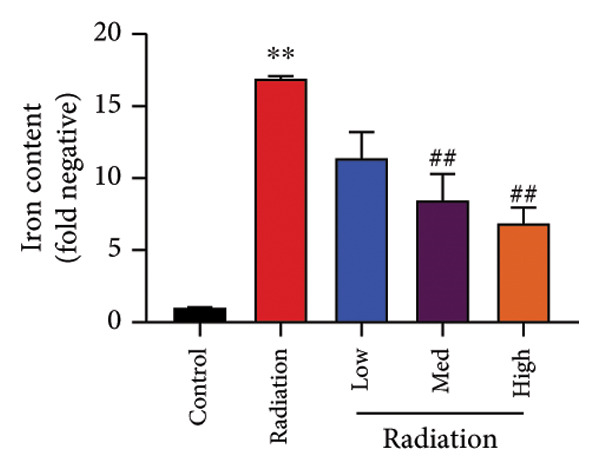
(d)

**Figure:**
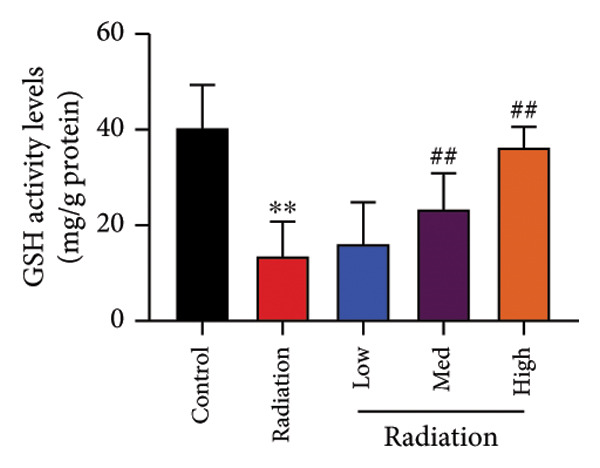
(e)

**Figure:**
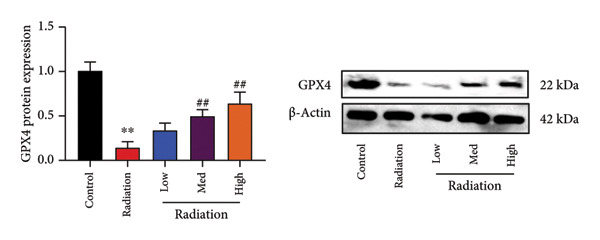
(f)

**Figure:**
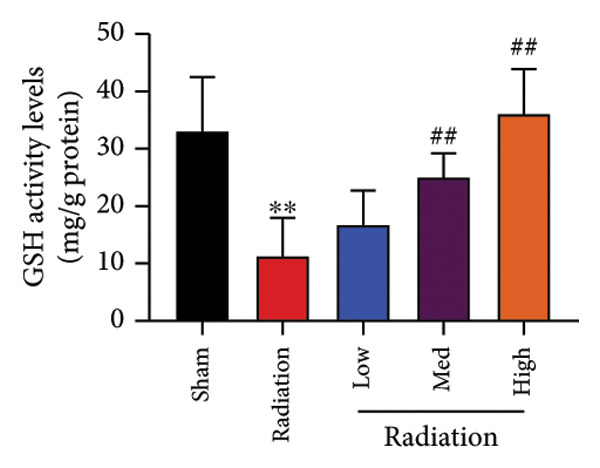
(g)

**Figure:**
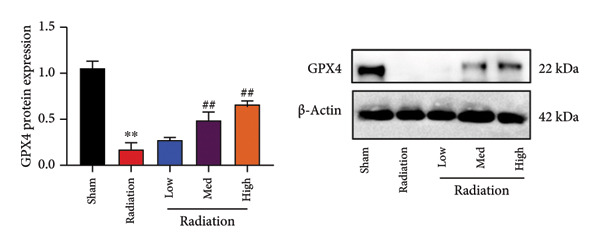
(h)

**Figure:**
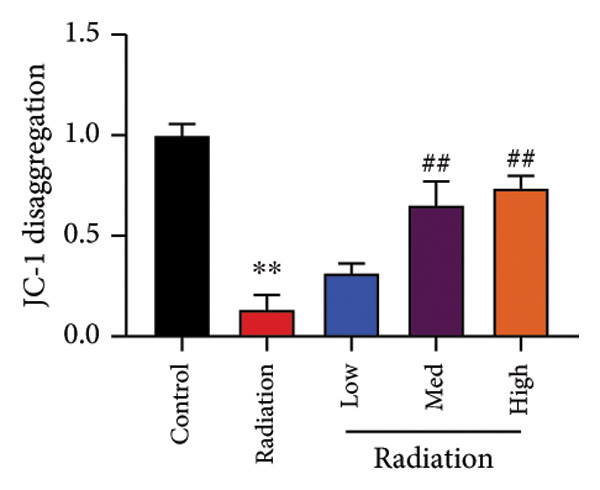
(i)

**Figure:**
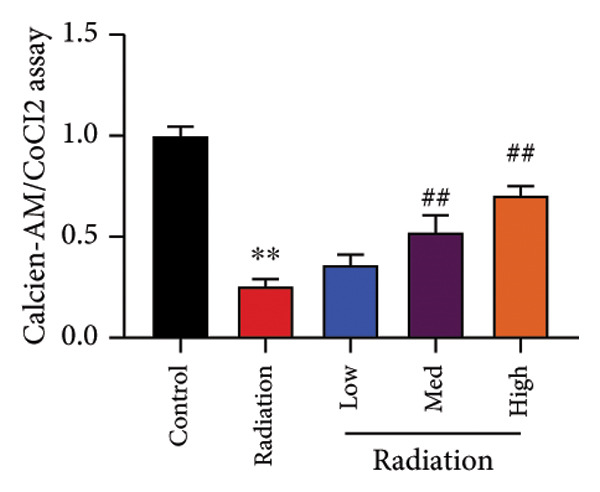
(j)

**Figure:**
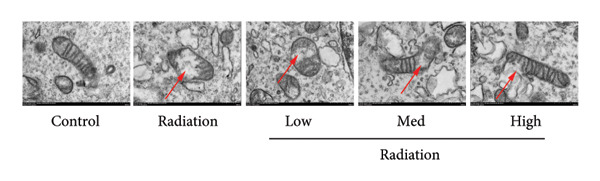
(k)

### 4.6. Nrf2 Inhibitor Reduced the Effects of Oroxylin A on Pulmonary Fibrosis in Model

The study further examined the effects of Oroxylin A on pulmonary fibrosis by Nrf2. Nrf2 inhibitor (2 mg/kg of Brusatol, 4 μg/mL of Brusatol) reduced the effects of Oroxylin A on Nrf2 protein expressions in the mice model or in the in vitro model (Figures 7(a), 8(a)). Nrf2 inhibitor also reduced the effects of Oroxylin A on pulmonary fibrosis in the mice model of RP (Figures 7(b), 7(c), 7(d), 7(e), 7(f), 7(g), 7(h), 7(i)). Nrf2 inhibitor inhibited the effects of Oroxylin A on inflammation, oxidative stress, and col3a1/FN/sma/twist mRNA expressions in the in vitro model of RP (Figures 8(b), 8(c), 8(d), 8(e)). Lastly, Nrf2 inhibitor also reduced the effects of Oroxylin A on ferroptosis in the in vitro model of RP (Figure 9).

Figure 7Nrf2 inhibitor reduced the effects of Oroxylin A on pulmonary fibrosis in the mice model. Nrf2/GPX4 protein expressions (a), inflammatory score (b), bronchiolar epithelium thickness (c), airway construction (d), central airway resistance (e), inspiratory capacity (f), quasi‐static compliance (g), col3a1/FN/SMA/twist mRNA expressions/pulmonary fibrosis (h), and IL‐1β/IL‐6/MDA/SOD/GSH‐PX (i) of lung tissue in the mice model. ^∗∗^ *p* < 0.01 compared with the control group.

**Figure:**
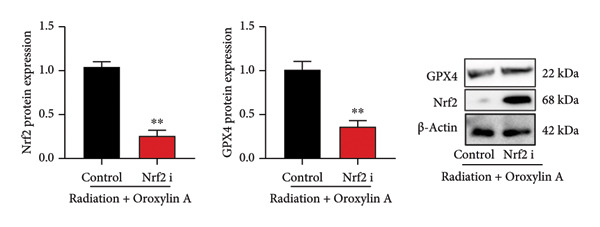
(a)

**Figure:**
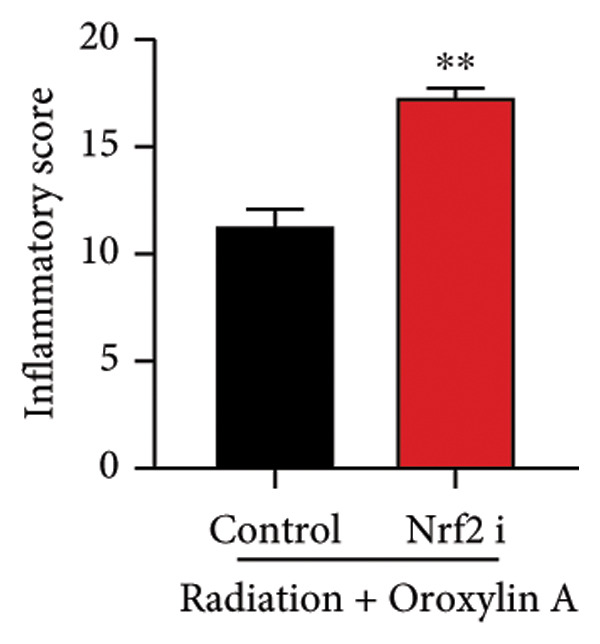
(b)

**Figure:**
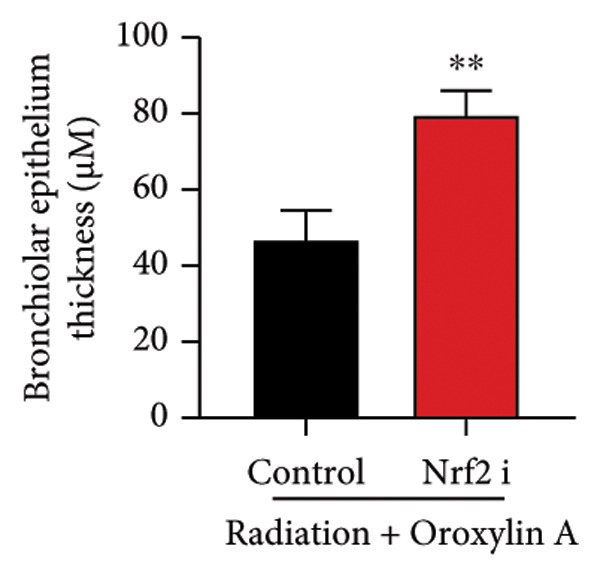
(c)

**Figure:**
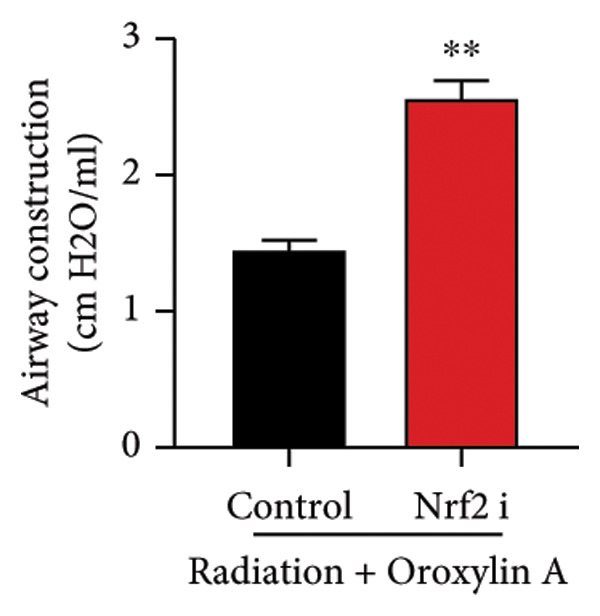
(d)

**Figure:**
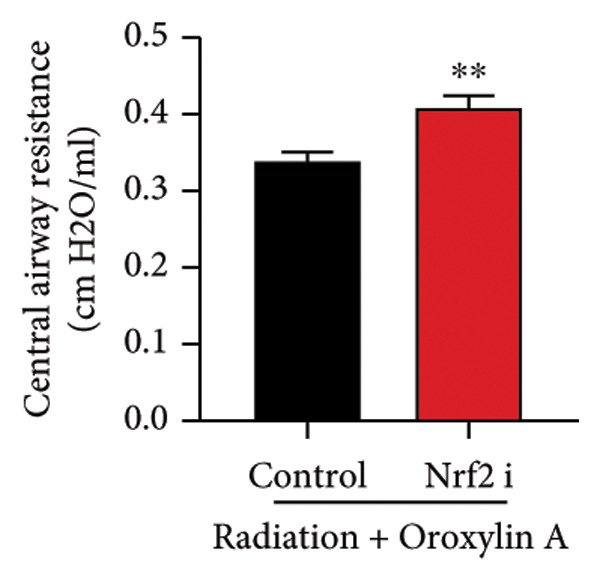
(e)

**Figure:**
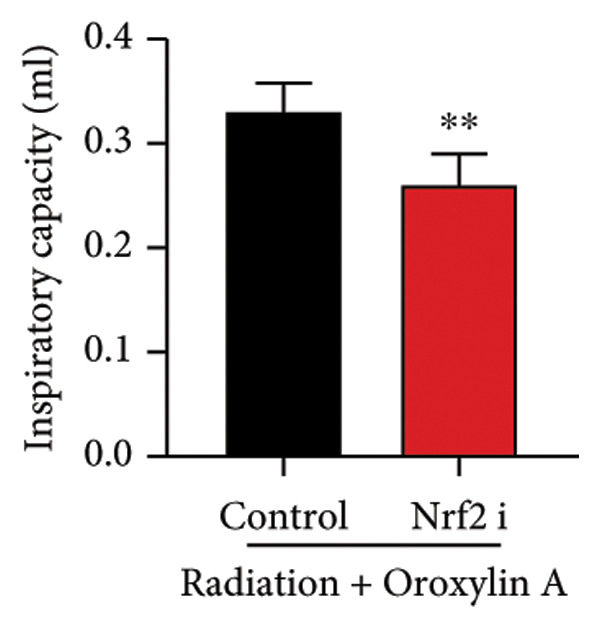
(f)

**Figure:**
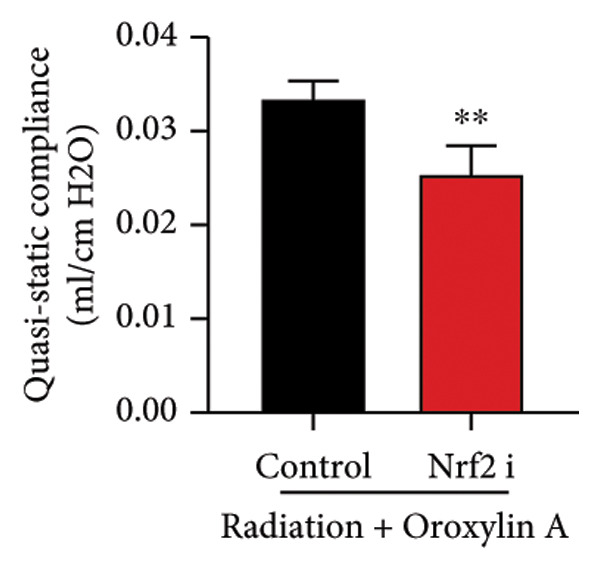
(g)

**Figure:**
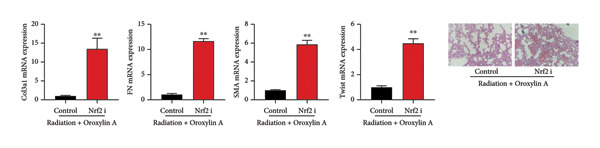
(h)

**Figure:**
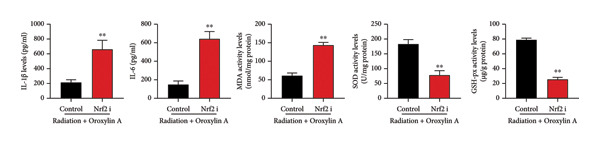
(i)

Figure 8Nrf2 inhibitor reduced the effects of Oroxylin A on pulmonary fibrosis in the in vitro model. Nrf2 protein expressions (a), IL‐1β/IL‐6 level (b), col3a1/FN/SMA/twist mRNA expressions (c), ROS/MDA (d), and SOD/GSH‐PX (e). ^∗∗^ *p* < 0.01 compared with the control group, ^##^ *p* < 0.01 compared with the radiation group, ^$$^ *p* < 0.01 compared with the Oroxylin A group.

**Figure:**
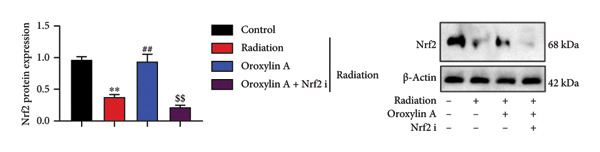
(a)

**Figure:**
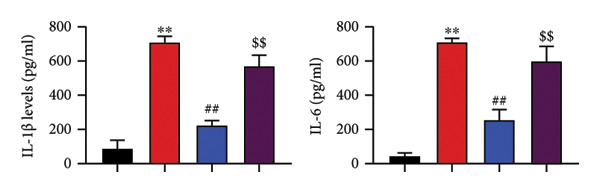
(b)

**Figure:**
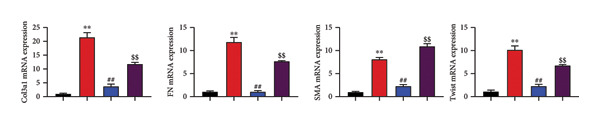
(c)

**Figure:**
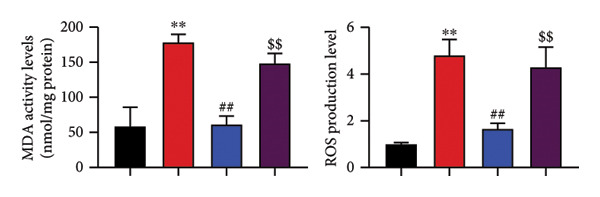
(d)

**Figure:**
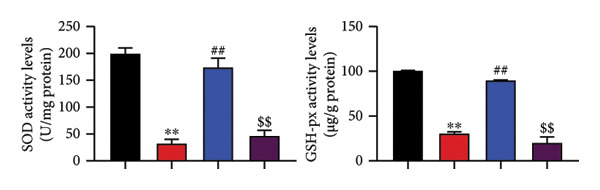
(e)

Figure 9Nrf2 inhibitor reduced the effects of Oroxylin A on pulmonary fibrosis in the in vitro model. Cell viability (a), LDH activity (b), PI rate levels (c), iron contents (d), GSH activity (e), GPX4 protein expressions (f), JC‐1 and AM/Cocl2 (i, j). ^∗∗^ *p* < 0.01 compared with the control group, ^##^ *p* < 0.01 compared with the radiation group, ^$$^ *p* < 0.01 compared with the Oroxylin A group.

**Figure:**
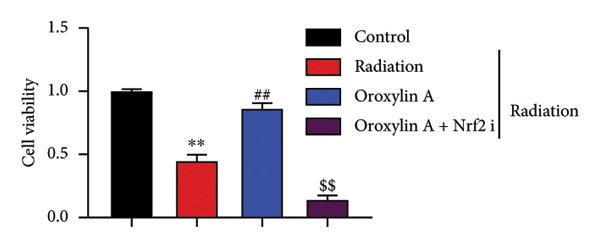
(a)

**Figure:**
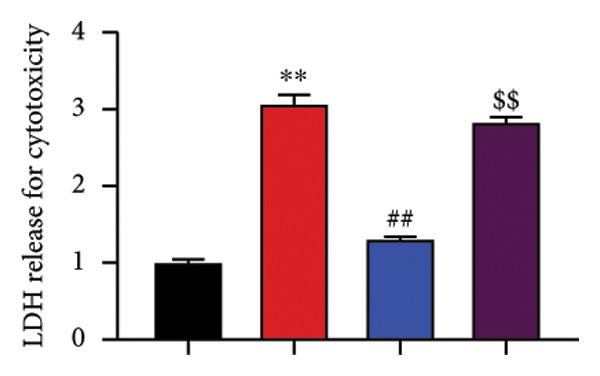
(b)

**Figure:**
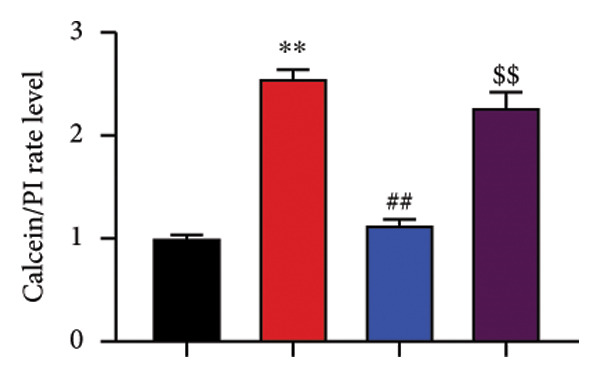
(c)

**Figure:**
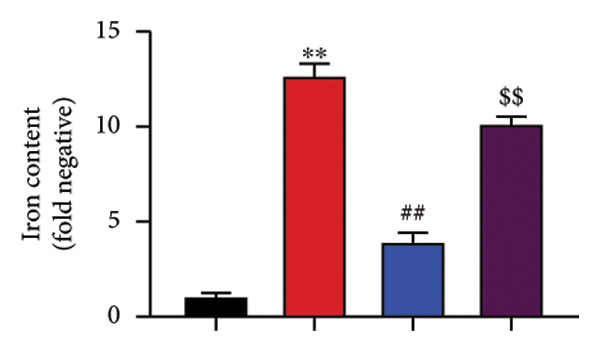
(d)

**Figure:**
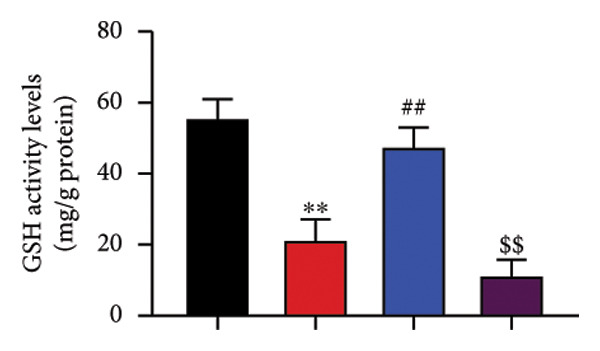
(e)

**Figure:**
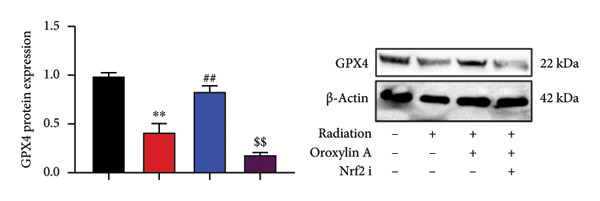
(f)

**Figure:**
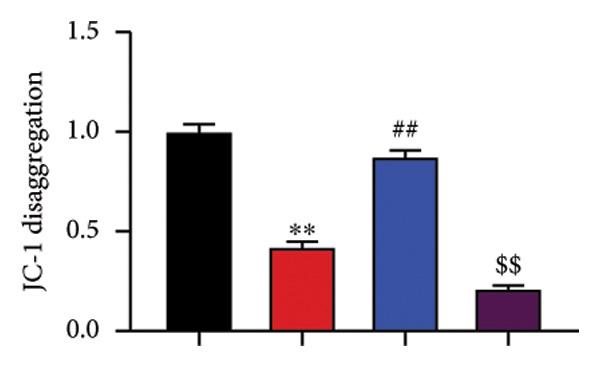
(g)

**Figure:**
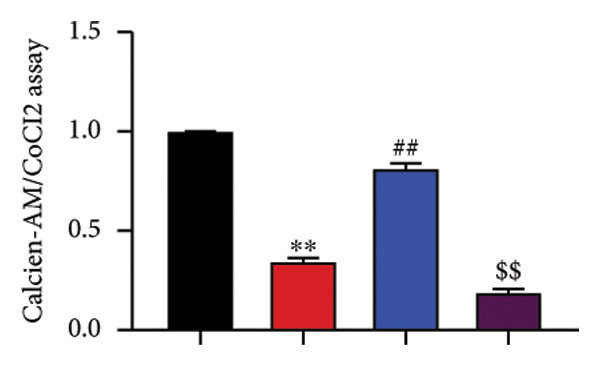
(h)

**Figure:**
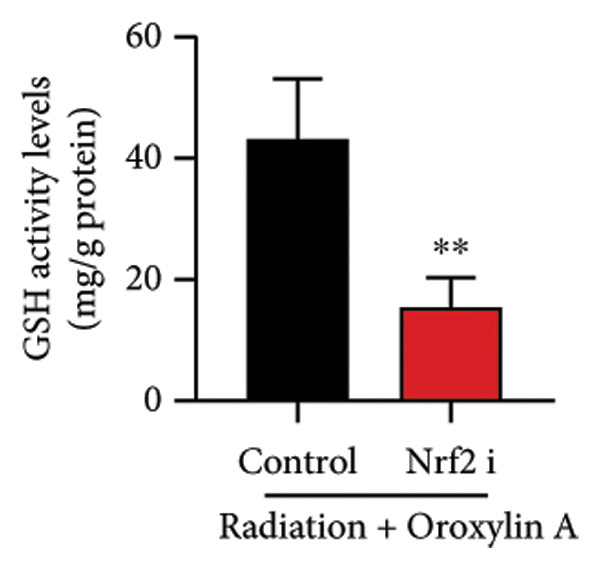
(i)

## 5. Discussion

Radiation‐induced pulmonary fibrosis (RIPF) literature indicates that the iron death pathway can be activated by inhibiting this signaling pathway [40–43]. However, with the global pandemic of COVID‐19, RIPF was manifesting as radiation pneumonitis or even severe pulmonary fibrosis [44]. This risk not only limits the radiation dose in clinical practice but also severely hinders patients’ quality of life, causing societal concern [45, 46]. In this study, we found the screening for an Oroxylin A from *Scutellaria baicalensis* for new Nrf2 agonists. Li et al. indicated that Oroxylin A induced the Nrf2 signaling pathway in the model of cigarette smoke [47]. Together, these data suggest that Oroxylin A might be used to prevent and cure pulmonary fibrosis of RP in further clinical treatment.

In Western medicine treatment, high‐dose antibiotics and hormone therapy are often used, but the adverse reactions are significant, the treatment duration is long, and hormone withdrawal syndrome, rebound phenomenon, bacterial resistance, etc., are prone to occur, resulting in long hospitalization periods, high costs, and more harmful sequelae after hormone therapy [48, 49]. Early diagnosis and treatment can protect lung function. Research has shown that the inflammatory background of the host affects the occurrence of radiation pneumonitis, such as the recruitment or local infiltration of T lymphocytes, which is associated with radiation pneumonitis and has become a common feature of radiation pneumonitis [16, 50]. Therefore, there is an urgent need for biomarkers that make it easy to evaluate the prognosis of radiotherapy patients. This results in iron death of alveolar epithelial cells, promoting fibroblast transformation and myofibroblast differentiation, which leads to excessive expression and deposition of ECM, ultimately causing fibrosis [24]. We found that Oroxylin A induced Nrf2 protein expression to reduce inflammation and oxidative stress in model of RIPF. Huang et al. showed that Oroxylin A inhibits inflammation in mice of carcinogen‐induced skin tumorigenesis [47]. Oroxylin A reduced inflammation and oxidative stress in model of RP.

Ferroptosis and GPX4 are closely related to infectious diseases such as pneumonia and pulmonary fibrosis, which may be an important factor leading to oxidative stress injury [51–53]. It not only causes organ and tissue damage by mediating cell death but also has a close relationship with inflammatory reaction, oxidative stress, etc. and can aggravate inflammatory and oxidative damage to tissues. GPX4 mediates the transport and internalization of iron into cells, initiating the process of cell iron death. GPX4 can alleviate lipid peroxidation and inhibit cell iron death [54–59]. However, many prospective therapeutic targets found in vitro or in animal models have not been applied in clinical trials [60]. It causes irreversible damage to the lungs. Currently, the main clinical approaches for treatment involve immunosuppressants in combination with corticosteroids, lung transplantation, or the use of drugs like pirfenidone and nintedanib to delay disease progression [61]. However, no highly effective and low side‐effect specific drugs have been discovered thus far [62]. Our study showed that Oroxylin A improved pulmonary fibrosis of RP in mice model. Sun et al. identified that Oroxylin A reduced hepatic fibrosis in hepatic stellate cell [63]. These findings support the conclusion that Oroxylin A improved pulmonary fibrosis of RP.

Oxidative stress is an important molecular mechanism underlying fibrosis in various organs, including the lungs [64]. It involves abnormal molecular, cellular, and tissue responses caused by ROS and/or depletion of antioxidant defense [49]. Ferroptosis, initially identified and proposed by Dixon, leads to iron deposition and subsequent cell death [65]. When cells undergo ferroptosis, a large amount of ROS is generated, exceeding the body’s clearance capacity, which results in an imbalance between oxidative and promoting pulmonary fibrosis [48]. This study found that Oroxylin A reduced ferroptosis in model of pulmonary fibrosis of RP.

Nrf2 is an important antioxidant stress element in the body, while Keap1 is an endogenous inhibitor of Nrf2 [11]. Only after Nrf2 dissociates from Keap1 it can enter the nucleus and exert its downstream antioxidant effects, producing HO‐1 to inhibit the ROS produced by iron death [10, 66, 67]. It is known that iron death cells release damage‐related molecular patterns and immune stimulatory cellular components, inducing cell death and inflammation. In addition, iron death of immune cells during infection can affect immune function and create a favorable environment for infectious factors. Downregulation of SLC7A11 expression can lead to reduced Gpx4 activity, decreased cellular antioxidant capacity, and increased ROS, ultimately resulting in ferroptosis. As an important transcription factor in oxidative reactions, Nrf2 has been reported to activate and regulate the metabolism of GSH, iron, and lipids, as well as mitochondrial function. SLC7A11 and Gpx4 are both regulated by Nrf2, while osthol can exert anti‐inflammatory activity by targeting and activating the Nrf2 antioxidant pathway. Our study found that Nrf2 inhibitor reduced Oroxylin A on pulmonary fibrosis in the model of RIPF. Therefore, it is speculated that GPX4 mediates Oroxylin A‐improved pulmonary fibrosis of RP through the induction of Nrf2.

**Figure 10:**
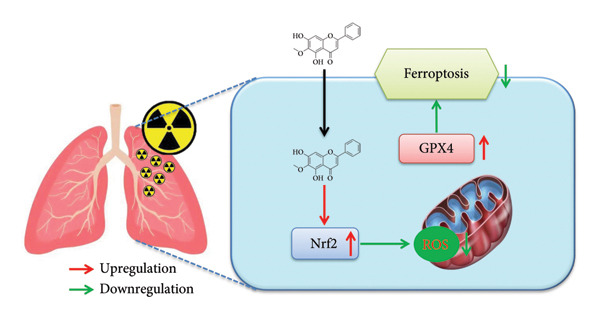
Oroxylin A from *Scutellaria baicalensis* improved pulmonary fibrosis of radiation pneumonia through ferroptosis by Nrf2.

In conclusion, Oroxylin A from *Scutellaria baicalensis* improved pulmonary fibrosis of RP through ferroptosis by the Nrf2 axle (Figure 10) and could serve as a potent therapeutic agent for alleviating RP.

**Nomenclature**
RP: Radiation pneumonia
EMT: Epithelial–mesenchymal transition
ECM: Extracellular matrix
Nrf2: Nuclear factor‐erythroid 2‐related factor‐2
GPX4: Glutathione peroxidase 4
MTT: 3‐(4,5‐Dimethylthiazol‐2‐yl)‐2,5‐diphenyltetrazolium bromide
qPCR: Quantitative polymerase chain reaction
ANOVA: One‐way analysis of variance
IL‐1β: Interleukin‐1 beta
IL‐6: Interleukin‐6
TNF‐α: Tumor necrosis factor‐alpha
ROS: Reactive oxygen species
MDA: Malondialdehyde
SOD: Superoxide dismutase
GSH: Glutathione
GSH‐px: Glutathione peroxidase

## Ethics Statement

The current study was approved by the Animal Ethics Committee and was conducted in accordance to the relevant agreements with The Affiliated Traditional Chinese Medicine Hospital, Southwest Medical University. All procedures were performed in accordance with the Guidance Suggestions for the Care and Use of Laboratory Animals, formulated by the Ministry of Science and Technology of China. All patients were informed and they signed informed consent voluntarily. This study was complied with the guidelines outlined in the Declaration of Helsinki, which were followed.

## Consent

Please see the Ethics Statement.

## Disclosure

All authors read and approved the final manuscript.

## Conflicts of Interest

The authors declare no conflicts of interest.

## Author Contributions

W.Z. and W.H. designed the experiments. X.G. and K.L. performed the experiments. W.Z. and W.H. collected and analyzed the data. X.G. and K.L. drafted manuscript.

## Funding

This study was supported by the Luzhou Science and Technology Program Project (No. 2022‐SYF‐95): Molecular Mechanism and Clinical Research on the Prevention and Treatment of Thoracic Tumor RP by Rhizoma Ligustici Chuanxiong and Scutellaria Baicalensis and the Compound Formula in the Yangtze River.

## Data Availability

The datasets used and/or analyzed during the current study are available from the corresponding author on reasonable request.
